# Supplementary material for: Cytocompatibility and Bioactive Ion Release Profiles of Phosphoserine Bone Adhesive: Bridge from In Vitro to In Vivo
Source: Biomedicines. 2022 Mar 22;10(4):736. doi: 10.3390/biomedicines10040736 (PMC9044752; doi:10.3390/biomedicines10040736)
Supplement: Supplementary file 1 [file biomedicines-10-00736-s001.zip › biomedicines-1624287-SI.pdf]

Supplementary material:

Table S1. Table of all disc formulations, including pH and setting time.

**Sample**	**P-ser**	**aTCP**	**CS-1**	**pH**	**cure time**	**Setting time (min)**	**aTCP**	**CS-1**	
Moles %	Moles %	Moles %	(min)	Initial	Final	Relative Moles %	Relative Moles %		
**1**	**26.5**	**42.3**	**31.2**	**7.098**	5	**57.5**	**42.5**		
**2**	**29.4**	**40.6**	**30.0**	5	**57.5**	**42.5**			
**3**	**27.8**	**34.9**	**37.3**	**7.000**	5	**48.4**	**51.6**		
**4**	**30.9**	**39.7**	**29.3**	**6.900**	5	**57.5**	**42.5**		
**5**	**31.8**	**42.8**	**25.4**	**6.452**	5	**62.8**	**37.2**		
**6**	**32.6**	**34.4**	**33.0**	**6.535**	5	**51.1**	**48.9**		
**8**	**27.0**	**18.3**	**54.7**	**7.450**	5	**25.1**	**74.9**		
**9**	**39.9**	**35.7**	**24.4**	**5.222**	5	**59.4**	**40.6**		
**10**	**37.6**	**30.2**	**32.2**	**6.479**	5	**48.4**	**51.6**		
**11**	**47.7**	**37.0**	**15.2**	**4.748**	5	**70.9**	**29.1**		
**13**	**24.7**	**49.1**	**26.2**	**7.017**	5	**65.2**	**34.8**		
**14**	**30.0**	**70.0**	**7.337**	5	**100.0**				
**15**	**40.0**	**60.0**	5	**100.0**					
**16**	**49.9**	**50.1**	**3.834**	5	**100.0**				
**18**	**34.0**	**10.6**	**55.5**	**7.313**	5	**16.0**	**84.0**		
**19**	**43.9**	**6.7**	**49.4**	**7.073**	5	**12.0**	**88.0**		
**20**	**25.9**	**32.5**	**41.6**	**7.188**	5	**43.9**	**56.1**		
**21**	**24.0**	**38.0**	**37.9**	**7.103**	5	**50.1**	**49.9**		
**22**	**14.0**	**49.8**	**36.2**	**7.600**	5	**57.9**	**42.1**		
**23**	**22.0**	**60.8**	**17.2**	**6.479**	5	**78.0**	**22.0**		
**24**	**18.0**	**73.9**	**8.1**	**6.227**	5	**90.1**	**9.9**		
**25**	**14.0**	**86.0**	**0.0**	**5.619**	5	**100.0**			
**26**	**0.0**	**100.0**	**7.193**	5	**100.0**				
**27**	**24.9**	**75.1**	**5.663**	1.6	1.6	3.5	**100.0**		
**28**	**28.8**	**39.7**	**31.6**	**7.409**	1	1	2.4	**55.7**	**44.3**
**29**	**35.1**	**61.6**	**3.4**	**5.771**	4.4	4.4	9.6	**94.8**	**5.2**
**30**	**23.3**	**20.9**	**55.8**	**7.936**	1	1	1.4	**27.3**	**72.7**
**31**	**24.0**	**12.1**	**63.9**	**7.974**	1	1	1.6	**15.9**	**84.1**
**32**	**30.7**	**29.2**	**40.1**	**7.609**	1	1	2	**42.1**	**57.9**
**33**	**42.4**	**26.1**	**31.5**	**6.270**	5.6	5.6	15.6	**45.3**	**54.7**
**34**	**25.0**	**75.0**	**7.527**	1	1	1.6	**100.0**		
**35**	**29.9**	**70.1**	**5.552**	2.4	2.4	7.2	**100.0**		
**36**	**69.9**	**30.1**	**2.875**	130	130	200	**100.0**		
**37**	**48.3**	**25.4**	**26.3**	**5.495**	26.8	26.8	120	**49.1**	**50.9**
**38**	**54.9**	**11.2**	**33.9**	**3.988**	16.8	16.8	46.5	**24.8**	**75.2**
**39**	**35.0**	**22.7**	**42.3**	**7.375**	1	1	2.8	**34.9**	**65.1**
**40**	**50.1**	**10.0**	**39.9**	**6.188**	6	6	24.5	**20.0**	**80.0**
**41**	**53.3**	**34.2**	**12.5**	**4.523**	59	59	122.5	**73.3**	**26.7**
**42**	**42.8**	**15.6**	**41.6**	**6.900**	1.8	1.8	9.2	**27.3**	**72.7**
**43**	**30.9**	**34.4**	**34.7**	**6.973**	5	**49.8**	**50.2**		
**44**	**50.0**	**12.5**	**37.4**	**5.160**	5	**25.1**	**74.9**		
**45**	**59.9**	**1.9**	**38.1**	**2.740**	5	**4.8**	**95.2**		
**46**	**23.3**	**34.8**	**41.9**	**7.419**	5	**45.4**	**54.6**		
**47**	**40.3**	**10.0**	**49.7**	**7.019**	5	**16.8**	**83.2**		
**48**	**36.8**	**27.0**	**36.2**	**6.111**	5	**42.8**	**57.2**		
**49**	**32.0**	**13.2**	**54.8**	**7.147**	5	**19.4**	**80.6**		
**50**	**29.9**	**22.3**	**47.7**	**7.230**	5	**31.9**	**68.1**		
**51**	**18.1**	**64.6**	**17.3**	**7.174**	5	**78.9**	**21.1**		
**52**	**28.3**	**54.9**	**16.8**	**5.910**	5	**76.5**	**23.5**		
**53**	**70.0**	**13.4**	**16.6**	**2.507**	5	**44.7**	**55.3**		
**54**	**75.0**	**5.0**	**20.0**	**2.285**	5	**20.2**	**79.8**		

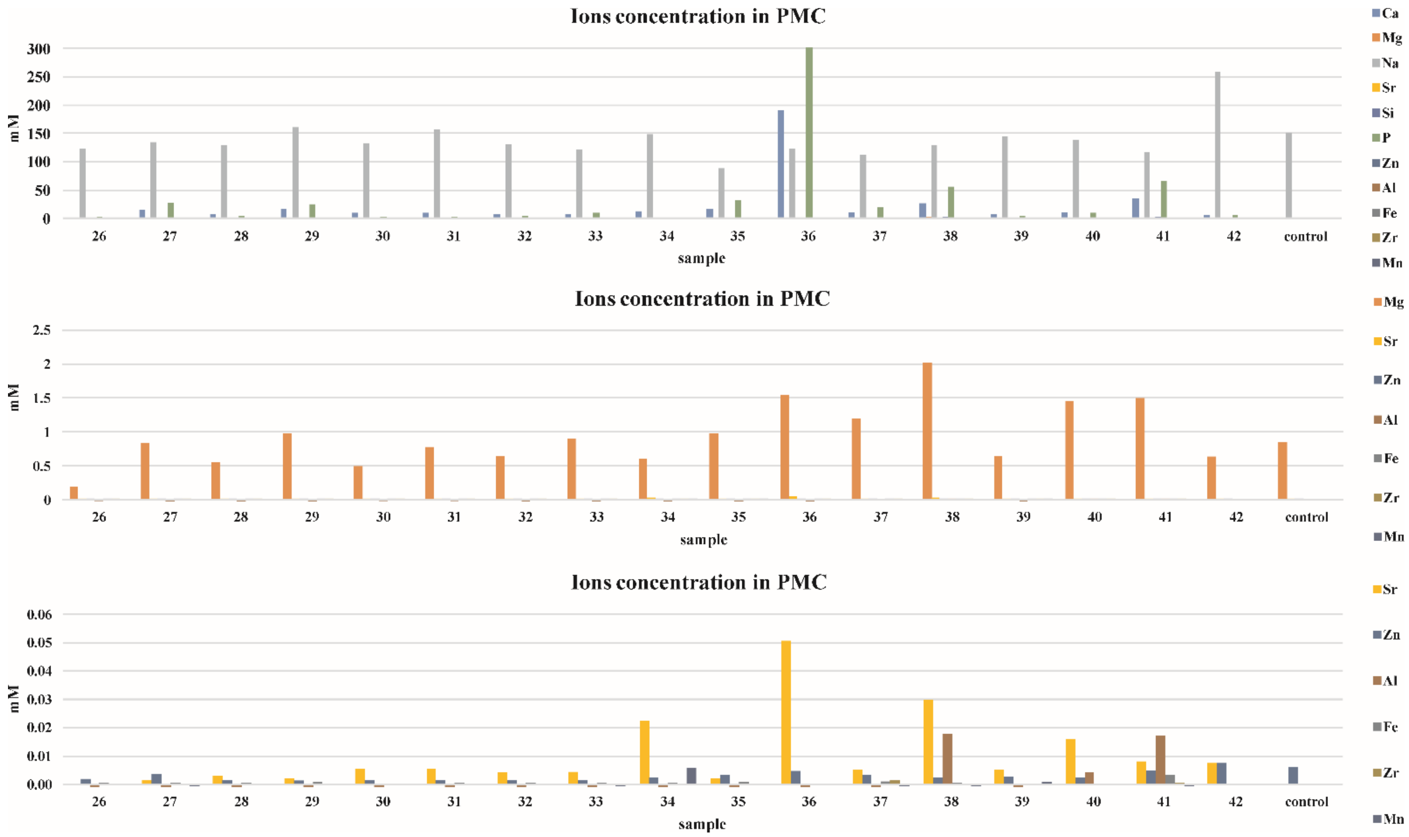

Supplementary Figure S1: The preliminary ICP data from the 17 different formulations

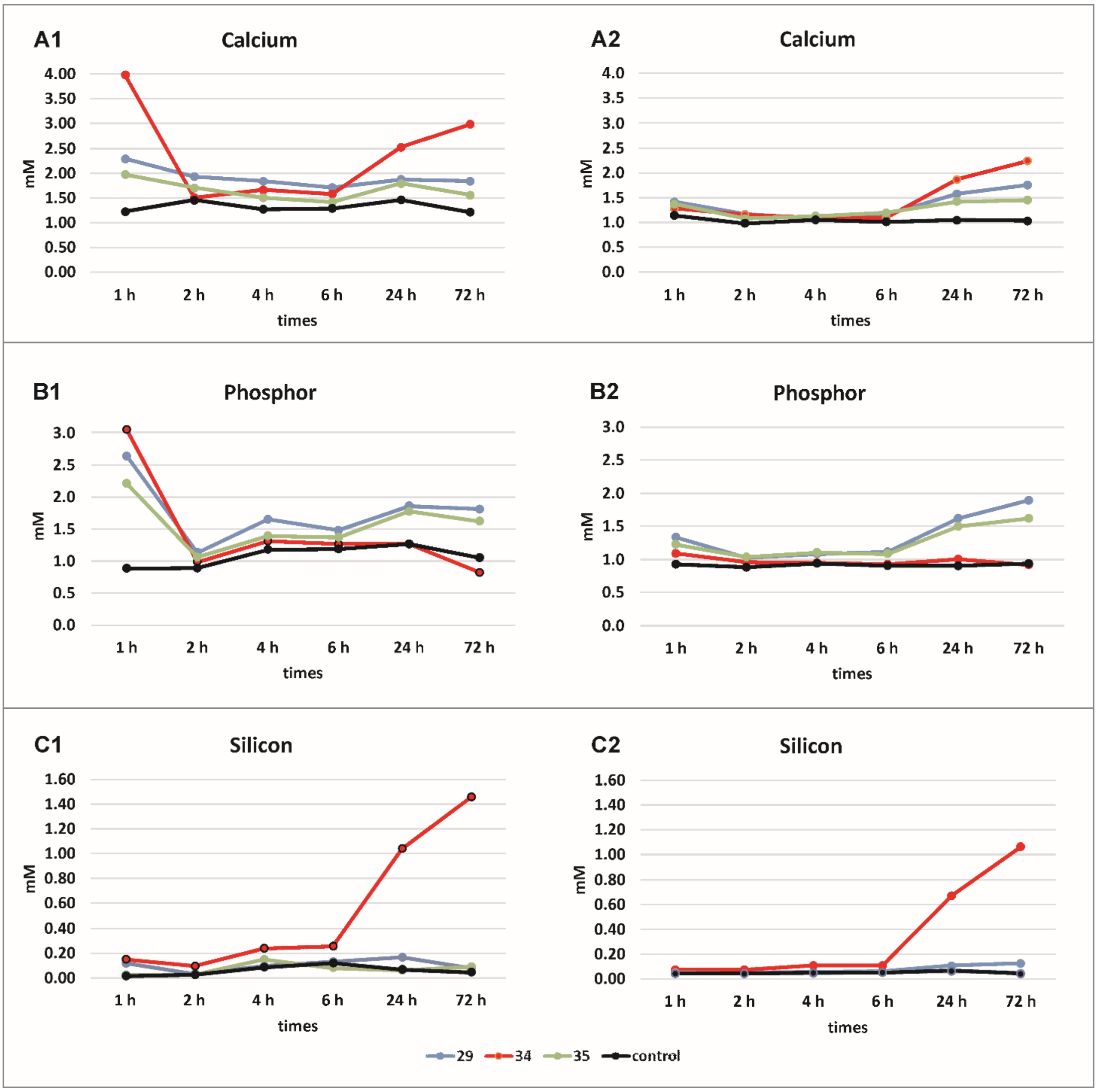

Supplementary Figure S2: Effect of curing time on ion release non-cumulative profiles. Non-cumulative ion release profiles for calcium (A), phosphorus (B), and silicon (C), for samples cured for the initial setting time (A1-C1) or 24 hours (A2-C2), via ICP-OES analysis. Sample dimensions were 1.6 mm × 1.6 mm (disc).

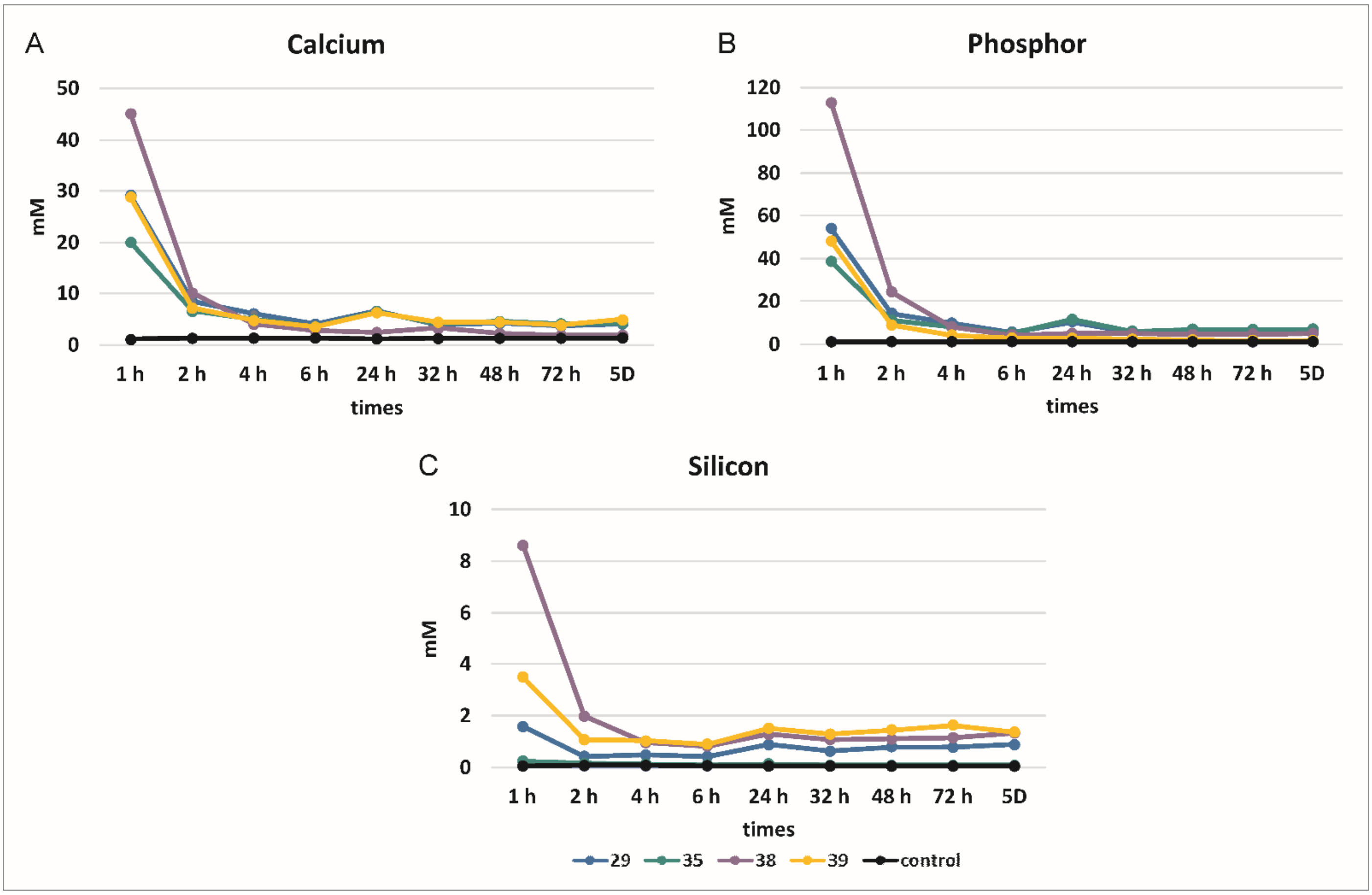

Supplementary Figure S3: Effect of media replenishment on ion release profiles, for a broad formulation range. Comparison of ion release non-cumulative profiles for (A) calcium, (B) phosphorus, and (C) silicon, from samples where the media was replenished at multiple time points ("dynamic"). Sample dimensions were 8.9 mm × 4.5 mm, cured to the initial setting time before immersion
